# Impact of a Rapid Diagnostic Meningitis/Encephalitis Panel on Antimicrobial Use and Clinical Outcomes in Children

**DOI:** 10.3390/antibiotics9110822

**Published:** 2020-11-18

**Authors:** Danielle McDonald, Christina Gagliardo, Stephanie Chiu, M. Cecilia Di Pentima

**Affiliations:** 1 Department of Pharmacy, Cooper University Health Care, Camden, NJ 08103, USA; danimcd2@gmail.com; 2 Department of Pediatrics, Goryeb Children’s Hospital, Atlantic Health System, Morristown, NJ 07960, USA; Christina.Gagliardo@atlantichealth.org; 3 Department of Pediatrics, Sidney Kimmel Medical College at Thomas Jefferson University, Philadelphia, PA 19107, USA; 4 Atlantic Center for Research, Atlantic Health System, Morristown, NJ 07960, USA; Stephanie.Chiu@atlantichealth.org; 5 Department of Pediatrics, Infectious Diseases Division, Atlantic Health System, Thomas Jefferson University, Morristown, NJ 07960, USA

**Keywords:** meningitis, encephalitis, FilmArray, multiplex PCR, antimicrobial, rapid diagnostic technology, stewardship, children, adolescents, outcomes

## Abstract

Rapid molecular diagnostic assays are increasingly used to guide effective antimicrobial therapy. Data on their effectiveness to decrease antimicrobial use in children have been limited and varied. We aimed to assess the impact of the implementation of the FilmArray Meningitis Encephalitis Panel (MEP) on antimicrobial use and outcomes in children. In an observational retrospective study performed at Atlantic Health System (NJ), we sought to evaluate the duration of intravenous antibiotic treatment (days of therapy (DoT)) for patients <21 years of age hospitalized and evaluated for presumptive meningitis or encephalitis before and after the introduction of the MEP. A secondary analysis was performed to determine if recovery of a respiratory pathogen influenced DoT. The median duration of antibiotic therapy prior to the implementation of the MEP was 5 DoT (interquartile range (IQR): 3–6) versus 3 DoT (IQR: 1–5) (*p <* 0.001) when MEP was performed. The impact was greatest on intravenous third-generation cephalosporin and ampicillin use. We found a reduction in the number of inpatient days associated with the MEP. In the regression analysis, a positive respiratory pathogen panel (RPP) was not a significant predictor of DoT (*p =* 0.08). Furthermore, we found no significant difference between DoT among patients with negative and positive RPP (*p =* 0.12). Our study supports the implementation of rapid diagnostics to decrease the utilization of antibiotic therapy among pediatric patients admitted with concerns related to meningitis or encephalitis.

## 1. Introduction

With the aid of rapid molecular diagnostics and the introduction of effective vaccines against *Haemophilus influenzae* type b, *Streptococcus pneumoniae*, and most recently *Neisseria meningitides*, the epidemiology of meningitis and encephalitis remains a rapidly evolving field [1]. The impact of vaccines has mainly affected children in developed countries, with an over 60% reduction in the incidence of bacterial meningitis in this patient population [2]. In a study performed in the United States in 2006, roughly 72,000 adult hospitalizations were related to meningitis [3]. While the majority of these were due to viral etiologies (54.6%), the estimated healthcare cost reached USD 1.2 billion [3]. More recent data show that the global incidence of meningitis increased from 2.5 million cases in 1990 to 2.82 million cases in 2016, with the highest rates found in sub-Saharan African countries, also known as the meningitis belt [4]. Kwambana-Adams et al. published the prevalence of bacterial, viral and parasitic infection in children younger than 5 years of age in West Africa following the rollout of conjugate vaccines against pneumococcus (PVC), meningococcus (MenAfriVac) and *Haemophilus influenzae* [5]. *Escherichia coli* (4.8%), followed by *S. pneumoniae* (3.5%) and *Plasmodium* (3.5%), were the most prevalent etiologies of meningitis in this age group. Because serotyping for pneumococcal isolates was not reported, the impact of PVC could not be determined. Gram negative rods, particularly *Escherichia coli* and *Klebsiella pneumoniae*, were more commonly identified in newborns.

The initial clinical manifestations of central nervous system (CNS) infections in neonates and children can be non-specific, difficult to diagnose and devastating if not treated correctly. The implementation of diagnostic stewardship entails optimization of clinical care and antimicrobial therapy guided by timely and personalized effective testing [6,7]. Rapid diagnostics have been shown to improve clinical outcomes in patients with bacteremia and infections with multidrug-resistant organisms when the introduction of these tests are linked to effective antibiotic stewardship strategies [7,8]. Data on the performance and impact of the FilmArray Meningitis Encephalitis Panel (MEP) in children are limited [9]. The MEP is a rapid multiplex polymerase chain reaction (PCR) assay designed to detect 14 pathogens in the cerebrospinal fluid (CSF). These pathogens include six bacteria, seven viruses, and one yeast group. In the cases of meningitis or encephalitis, quick pathogen identification aids in the initiation/continuation of appropriate targeted therapy as well as discontinuation of unnecessary empiric antimicrobials. Timely diagnosis directly impacts patient outcomes and healthcare costs.

Prior to Food and Drug Administration (FDA) approval of the MEP, a large, prospective, multicenter study of 1560 CSF specimens was conducted to compare the MEP to standard diagnostics, bacterial culture and viral PCR [10]. In this study, the MEP yielded a percent positive agreement (PPA) of 100% for 9 of 14 analytes. Enterovirus yielded a 95.7% PPA, and human herpes virus type 6 had an agreement of 85.7%. *Streptococcus agalactiae* had one false-positive and one false-negative result. *Listeria monocytogenes* and *Neisseria meningitides* were not evaluated.

Additional studies augmented the results and strengthened the findings of this initial study. Liesman et al. evaluated 291 CSF specimens and found a PPA of 85.6% [11]. When results for *Cryptococcus neoformans/gattii* were excluded, the PPA increased to 92.5%. Naccache et al. evaluated 251 samples and showed a low false positivity rate [12]. Piccirilli et al. demonstrated 90.9% concordance between the FilmArray MEP and conventional microbiological procedures in 77 CSF samples studied [13]. Additionally, two published reviews had a pediatric focus. Graf et al. used 67 retrospective viral PCR or bacterial culture-positive samples and identified 92.5% that were positive for the same target on the panel [14]. Messacar et al. tested 138 CSF samples and concluded an overall agreement of 96% as compared to conventional diagnostic methods in children with CNS infections [15]. In a recently published meta-analysis by Tansarli and Chapin, and as previously reported by Liesman et al., the MEP was found to have higher rates of false-negative results for herpes simplex virus 1 and 2 and enterovirus when compared with standard PCR assays [11,16].

More recently, several studies demonstrated cost savings and reductions in antibiotic days of therapy (DoT) with implementation of the MEP [17,18,19,20,21]. Nabower et al. demonstrated decreased length of stay (LOS) and fewer acyclovir doses administered, while Weber et al. demonstrated hospital cost savings in a military treatment facility [17,18]. Posnakoglou et al. supplemented these findings, demonstrating decreased LOS, a reduction in antimicrobial use, and a decrease in total cost [19]. Similarly, Hagen et al. noted a decreased duration of empiric therapy, with the largest effect documented in infants [20]. Messacar et al. focused on herpes simplex virus in patients >60 days of age and observed a doubling of herpes simplex virus testing with a reduction in acyclovir duration of therapy [21]. These studies begin to validate the clinical utility of the MEP in pediatric patients with results that support opportunities for antimicrobial stewardship.

The purpose of this study was to evaluate the impact of the implementation of the FilmArray MEP in pediatric patients receiving empiric therapy for meningitis and/or encephalitis. The potential confounding role of respiratory pathogens was examined in a secondary analysis.

## 2. Methods

In an observational retrospective study performed at Atlantic Health System (AHS), we reviewed 297 medical records of patients <21 years of age evaluated for meningitis and/or encephalitis between January 2015 and September 2018. AHS is a not-for-profit private healthcare corporation operating five hospitals in northern New Jersey. Subjects evaluated at two AHS hospitals were included in the study: Goryeb Children’s Hospital in Morristown and Goryeb Children’s Center at Overlook Medical Center in Summit.

Admitted patients evaluated for meningitis by lumbar puncture prior to MEP incorporation were categorized and analyzed as “pre-implementation” subjects (January 2015–October 2016), whereas admissions on or after incorporation were categorized as “post-implementation” subjects (November 2017–September 2018). In order to only assess duration of empiric therapy, patients with confirmed bacterial infections and patients with herpes simplex meningoencephalitis were excluded from the study. Confirmation was based on positive MEP and CSF, blood cultures, and urine cultures. Hematology–oncology and neurosurgery patients were also excluded. A total of 247 patients were included in the final analysis.

The FilmArray MEP (BioFire Diagnostics, Salt Lake City, UT, USA) was incorporated at AHS on November 1, 2016. Analytes on the MEP include *Escherichia coli* (K1 capsular type), *Haemophilus influenzae*, *Listeria monocytogenes*, *Neisseria meningitides*, *Streptococcus agalactiae*, *Streptococcus pneumoniae*, *Cytomegalovirus*, *Enterovirus*, *Epstein-Barr virus*, *Herpes simplex viruses 1 and 2*, *Human Herpes virus 6*, *Varicella zoster*, *Human parechovirus*, and *Cryptococcus neoformans/gattii*. The sample size required is 200 microliters (μL), and the turnaround time for reporting the MEP at AHS is approximately 2 h. FilmArray Respiratory Pathogen panel (RPP; BioFire Diagnostics, Salt Lake City, UT, USA) was incorporated in 2011. Analytes of the RPP include Adenovirus, Coronaviruses (HKU1, NL63, 229E, OC43), Human metapneumovirus, Human rhinovirus/Enterovirus, Influenza A (A/H1, A/H#, A/H1-2009) and Influenza B viruses, Parainfluenza (1–4) viruses, Respiratory Syncytial Virus, *Bordetella pertussis*, *Bordetella parapertussis*, *Chlamydia pneumonia*, and *Mycoplasma pneumoniae*.

Data collected included patient age, gender, admission date, event date, CSF studies, diagnosis, antimicrobial therapy, RPP if performed, mortality and 30-day readmission.

The primary outcome of the study was to evaluate the duration of empiric antimicrobial therapy measured as DoT before and after incorporation of the MEP. Secondary outcomes included length of stay (LOS), all-cause mortality and 30-day readmission rates. Patient outcomes were compared pre- and post-implementation.

## 3. Statistical Analysis

Patient characteristics were summarized using medians and interquartile range (IQR) for continuous variables and proportions for categorical data. Total DoT and LOS failed normality tests, so non-parametric comparative analyses, Mann–Whitney, were performed to assess the data between the two groups. Binomial variables were compared using 2 proportions, and binary regression analyses were used to determine significant predictor variables. Categorical variables were evaluated using a chi-square test. All tests were 2-tailed at a level of significance of less than 0.05.

The AHS institutional review board approved this study (Protocol Number: 1107015-1).

## 4. Results

Two-hundred and forty-seven children with suspected meningitis or encephalitis who received empiric antimicrobial therapy were included in the study analysis. Of these, 186 patients were part of the pre-implementation period while 61 patients had an MEP performed during the post-implementation period. Patient characteristics for each group are depicted in Table 1. Age and gender were similar in both groups. The median age for all patients was less than 1 year of age. A total of 113 (60%) and 37 (64%) patients were males before and after the implementation of the MEP, respectively. Even when a higher proportion of patients was admitted to intensive care units during the pre-implementation phase, this difference was not statistically significant (*p =* 0.16). Patients were more likely to have a positive RPP prior to the implementation of the MEP (*p <* 0.01).

**Figure 1:**
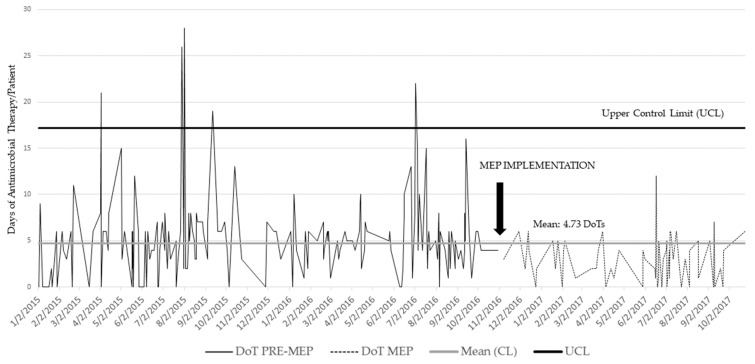
Patient-specific empiric antimicrobial utilization.

The median duration of antibiotic therapy in the pre-implementation group was five DoT (IQR: 3–6) versus three DoT (IQR: 1–5) (*p <* 0.001) post–implementation. Figure 1 illustrates antibiotic utilization before and after the MEP was introduced into clinical practice in our study population.

During the pre-implementation period, the median DoT for individual antibiotics was 3 DoT (IQR: 1) for third-generation cephalosporins, including ceftriaxone and cefotaxime (*n*:23), 3 DoT (IQR: 3) for ampicillin (*n*:113) and 2 DoT (IQR: 2–3) for vancomycin (*n*:40). Ceftazidime was not used in either cohort.

The median duration of empiric antibiotic therapy in patients with suspected meningitis or encephalitis during the post-implementation period was 2 DoT (IQR: 2–3) for third-generation cephalosporins (*n*:31) (*p =* 0.02), 2 DoT (IQR: 2–3) for ampicillin (*n*:22) (*p =* 0.017) and 2 DoT (IQR: 2–3) for vancomycin (*n*:7) (*p* = 0.2). Gentamicin was used in 65 and 17 subjects, before and after the implementation of the MEP with a median duration of 2 DoT for both patient groups (*p =* 0.13). We found no statistical differences in the median duration of cefepime (*p =* 0.70), doxycycline (*p =* 0.9) or piperacillin-tazobactam (*p =* 0.95) between the two cohorts.

Few patients received acyclovir before (*n*:32) and after implementation of the MEP (*n*:9). Median utilization of acyclovir was 3 DoT (IQR: 3–4) and 2 DoT (IQR: 2–3), respectively (*p =* 0.76).

In the regression analysis, in patients evaluated for meningitis or encephalitis, a positive RPP was not a significant predictor of duration of antibiotic therapy (Odds Ratio: 1.15; 95% Confident Interval: 0.1–1.34). Furthermore, we found no significant differences between DoT among patients with negative (median: 4 DoT; range 0–6) and positive (median: 4 DoT; range 0–21) RPP (*p =* 0.12).

Secondary outcomes are summarized in Table 2. We found a statistically significant reduction in the median number of inpatient days after the implementation of the MEP (*p <* 0.01). All-cause readmission was higher in the pre-implementation group but did not reach statistical significance (*p =* 0.24). No deaths occurred in either cohort.

## 5. Discussion

In our experience, implementation of the MEP decreased antimicrobial use and LOS among hospitalized children evaluated for presumptive meningitis or encephalitis, without having a negative impact on readmissions or mortality.

Performing a lumbar puncture in young infants and children can be challenging, limiting the ability to obtain large volumes of CSF to submit for multiple tests, especially when standard antigen, PCR and/or antibody testing must be performed at different reference laboratories. The MEP uses only 200 μL of CSF. Reference laboratories usually request a minimum of 500 μL of CSF to perform individual pathogen testing such as *Cryptococcus* antigen, HSV or enterovirus PCRs. CSF culture, although still the gold standard for diagnosis, takes a longer time to result. Standard microbiological methods for recovery and identification of an organism can take up to 48–72 h to report, and turnaround times for reference laboratories mean that it can take days to deliver results. At our institution, the MEP is reported within 2 h of obtaining the CSF sample. Furthermore, culture results can be difficult to interpret in patients who previously received antibiotic treatment. In a small study of 62 CSF samples from young infants with suspected meningitis, seven samples were positive on the PCR panel with no culture growth [22]. These seven samples were obtained from infants who had been pretreated with antibiotics. While false-positive and false-negative results from the MEP are possible, and results need to be interpreted in the context of the patient’s clinical condition, the MEP may increase the ability to recover a clinically significant organism in children who have been pretreated with antimicrobials.

We noted that impact on antibiotic utilization mainly affected intravenous ampicillin, commonly used in newborns with suspected early or late onset sepsis and/or meningitis when *Streptococcus agalactiae*, *Listeria monocytogenes*, *E. coli*, and other Gram-negative pathogens are a consideration. With rates of ampicillin-resistant *E. coli* surpassing 50%, early identification of a potential etiology can guide appropriate antibiotic therapy. Similar impact was noted on third-generation cephalosporins, the antibiotics of choice for empiric therapy for infants and children with suspected CNS infections. Despite the intermittent shortages and eventual discontinuation of cefotaxime, and the age limitations for the use of ceftriaxone during the newborn period, we did not find a statistically significant change in the use of cefepime. Ceftazidime, an alternative to cefotaxime recommended by the American Academy of Pediatrics for infants under 2 months of age with suspected meningitis, was not used in our patient population. While the duration of empiric gentamicin use did not change with the rollout of the MEP, fewer patients were started on this antibiotic after the MEP was implemented.

The impact found on antibiotic utilization was independent of patients diagnosed with a respiratory viral pathogen. Furthermore, a positive RPP was not associated with a shorter duration of antibiotic therapy, implying that diagnosis of a viral respiratory infection did not drive antibiotic management in this patient population. Studies assessing the impact of rapid diagnostics in children with acute respiratory tract infections found that these assays reduce LOS and empiric antibiotic utilization [23]. To the best of our knowledge, prior studies assessing the impact of RPP in children admitted with possible CNS infection has not been published. Our data suggest that rapid syndromic molecular testing has a more meaningful impact when aimed at the diagnosis of concern rather than in combination.

Herpes simplex virus can be a devastating CNS infection in newborns. Although rare, with rates in the United States ranging between 1 in 2000 to 1 in 13,000 live births, early diagnosis and treatment remain critical to impact mortality and neurologic outcomes in this patient population [24]. In our institution, prior to the implementation of the MEP, CSF herpes simplex virus DNA testing was sent out to an outside laboratory, delaying turnaround time by several days. Though acyclovir use decreased after the implementation of the MEP, the small sample size made it difficult to assess the statistical impact on acyclovir DoT. However, rapid negative herpes simplex virus results from the MEP reduced the number of patients receiving empiric acyclovir therapy for several days pending results, as was seen in the pre-implementation period. These findings are critically important in the phase of recurrent shortages of intravenous acyclovir. Acyclovir shortages trigger the need to use alternative therapies such as IV ganciclovir or high-dose oral valacyclovir [25]. Of particular concern is the potentially negative economic and clinical impact associated with drug shortages described in the literature [26]. As of 22 September 2020, acyclovir remains in the drug shortage list kept by the American Society of Health-Systems Pharmacists [27]. The COVID-19 pandemic has generated an additional challenge to the chronic problem of antimicrobial shortages by creating an imbalance between supply and demand [28]. Furthermore, it is estimated that the global public health crisis generated by the severe acute respiratory syndrome COVID-19 resulted in an increased use of antimicrobials and could amplify the threat of antimicrobial resistance [29].

There are limitations to this study. We excluded hematology, oncology and neurosurgery patients. However, we purposely excluded these populations, in whom empiric antibiotic therapy might be guided based on risks associated with their underlying conditions. We also excluded hospitalized patients with documented infections to assess only the duration of empiric antimicrobial therapy. Our patient population represents a single institution, and the results might not be generalizable to all centers caring for children. Our study did not include an economic analysis. Duff et al. evaluated the financial outcome of the implementation of the BioFire^®^ MEP in adult and pediatric populations at a single institution [30,31]. Greater savings were found when testing was performed in all suspected cases rather than in those with abnormal CSF findings for both pediatric (USD 3481/case) and adult (USD 2213/case) patients [30,31]. While antibiotic use is known to be associated with the emergence of antibiotic resistance, the direct impact of a single rapid diagnostic test is difficult to discern. To the best of our knowledge, studies evaluating the potential impact of the MEP on antibiotic resistance has not been published.

The World Health Organization is committed to decreasing the scourge of bacterial meningitis, especially for pathogens affecting young children, such as *Streptococcus agalactiae*, *Neisseria meningitidis*, *Streptococcus pneumoniae*, and *Haemophilus influenzae* [32]. The “Defeating meningitis by 2030” global roadmap is a multi-organization partnership that calls for the development and wide implementation of molecular-based multiplex meningitis rapid diagnostic assays at the point of care [32]. Newer generation meningitis assays will be needed to accomplish these goals worldwide [33]. Despite all the benefits described, rapid diagnostic tests should not replace routine bacterial and fungal cultures. Moreover, diagnostic testing should be interpreted in the context of the patient’s clinical manifestations, and the possibility of either a false-positive or false-negative result should be considered based on the index of clinical and laboratory suspicion.

## 6. Conclusions

Meningitis remains a prevalent and devastating disease worldwide. While the timely administration of antimicrobial therapy is critical to optimizing the outcomes of children with CNS infections, unnecessary treatments and prolonged hospitalizations represent a burden on our healthcare system and contribute to antimicrobial shortages and resistance. In our experience, the implementation of rapid CSF multiplex PCR assays aided in antimicrobial stewardship initiatives and shortened the duration of hospital stays in children with suspected meningitis and encephalitis.

## Figures and Tables

**Table 1:** Patient Characteristics.

Characteristics	Pre-MEP (N:186)	Post-MEP (N:61)	*p*-Value
Age in years, median (IQR)	0 (0–3.5)	0 (0–4)	0.24 ^†^
Male patients, n (%)	113 (59.8)	37 (63.7)	0.59 *
NICU/PICU care, n (%)	43 (23)	20 (32)	0.16 *
CSF WBC, median (IQR)	4 (1–22.3) cells/mm^3^	3 (1–13.5) cells/mm^3^	0.71 ^†^
Respiratory pathogen panel positive n (%)	105/109 (96.3)	17/40 (42.5)	<0.01 *

^†^ Mann–Whitney, * chi-square test, IQR: interquartile range, NICU: neonatal intensive care unit, PICU: pediatric intensive care unit, CSF: cerebrospinal fluid, WBC: white blood cells.

**Table 2:** Patient Outcomes.

Outcomes	Pre-MEP (N:186)	Post-MEP (N:61)	*p*-Value
LOS, median (IQR)	4 (1–3)	3 (0–4)	<0.001 ^†^
All-cause 30 day-readmission (%)	4 (2.2%)	0	0.24 *
All-cause mortality	0	0	

^†^ Mann–Whitney, * chi-square test.
